# Supplementary material for: Empirical methods for controlling false positives and estimating confidence in ChIP-Seq peaks
Source: BMC Bioinformatics. 2008 Dec 5;9:523. doi: 10.1186/1471-2105-9-523 (PMC2628906; doi:10.1186/1471-2105-9-523)
Supplement: Additional File 1 — A variety of html documents from the USeq web site detailing the available applications, their best usage, output file type descriptions, command line menus, etc. [file 1471-2105-9-523-S1.zip › USeqUserGuides/JavaDoc.zip!/JavaDoc/trans/tpmap/class-use/IntensityFeature.html]

Uses of Class trans.tpmap.IntensityFeature


|  |  |  |
| --- | --- | --- |
| **Overview** \| **Package** \| **Class** \| **Use** \| **Tree** \| **Deprecated** \| **Index** \| **Help** | |  |
| PREV   NEXT | **FRAMES**    **NO FRAMES**     **All Classes** |  |


---


## **Uses of Class trans.tpmap.IntensityFeature**

| Packages that use IntensityFeature | |
| --- | --- |
| **trans.cel** | Apps and methods for manipulating cel files. |

Uses of IntensityFeature in trans.cel

| Methods in trans.cel that return IntensityFeature | |
| --- | --- |
| `static IntensityFeature[]` | `MakeChromosomeSets.collapseIntensityFeatureArray(IntensityFeature[][] fs)`             Collapses an uneven array of IntensityFeature[][] to IntensityFeature[]. |
| `static IntensityFeature[]` | `MakeChromosomeSets.fetchIntensityFeatures(java.io.File[] f)`             Loads IntensityFeature[]s into a single master. |

| Methods in trans.cel with parameters of type IntensityFeature | |
| --- | --- |
| `static boolean` | `CelProcessor.anyNaNs(IntensityFeature[] ints)`             Looks for NaN in IntensityFeatures. |
| `static IntensityFeature[]` | `MakeChromosomeSets.collapseIntensityFeatureArray(IntensityFeature[][] fs)`             Collapses an uneven array of IntensityFeature[][] to IntensityFeature[]. |
| `static void` | `MakeChromosomeSets.splitAndSaveIntensities(IntensityFeature[] ints, java.io.File f)`             Saves the intensities to file as a int[] |
| `static void` | `MakeChromosomeSets.splitAndSavePositions(IntensityFeature[] ints, java.io.File f)`             Saves the positions to file as a int[] |

---


|  |  |  |
| --- | --- | --- |
| **Overview** \| **Package** \| **Class** \| **Use** \| **Tree** \| **Deprecated** \| **Index** \| **Help** | |  |
| PREV   NEXT | **FRAMES**    **NO FRAMES**     **All Classes** |  |


---
